# The status and predictors of self‐care among older adults with hypertension in China using the Chinese version of Self‐Care of Hypertension Inventory – A cross‐sectional study

**DOI:** 10.1002/nop2.1165

**Published:** 2022-01-11

**Authors:** Yu‐Jie Guo, Xiao‐Yun Hu, Hong‐Juan Ji, Qiao Zhao, Long‐Yuan Wang, Xiao‐Yan Zhou, Jue Tang, Lei Yang, Xiang‐Chao Sun

**Affiliations:** ^1^ School of Medicine (School of Nursing) Nantong University Nantong China; ^2^ 12461 School of Pharmacy Nanjing Medical University Nanjing China; ^3^ Department of Rehabilitation Affiliated Hospital of Nantong University Nantong China; ^4^ Department of Nursing Wuxi Higher Health Vocational Technology School Wuxi China; ^5^ Second School of Clinical Medicine Nanjing Medical University Nanjing China; ^6^ Department of Internal Medicine Third People’s Hospital of Rugao Nantong China; ^7^ First School of Clinical Medicine Nanjing Medical University Nanjing China

**Keywords:** hypertension self‐care, older adults, predictors, Self‐care of Hypertension Inventory (SC‐HI)

## Abstract

**Aim:**

To investigate the status and predictors of self‐care among older adults with hypertension in China by the Chinese version of Self‐Care of Hypertension Inventory.

**Design:**

A cross‐sectional questionnaire survey.

**Methods:**

A convenience sampling of 544 older adults with hypertension was surveyed using the Chinese version of Self‐Care of Hypertension Inventory. SPSS25.0 software was used for statistical analysis of the data. Generalized liner model univariate analysis and the optimal scaling regression analysis were performed to investigate the predictors of self‐care.

**Results:**

The status of self‐care was poor with the median and inter‐quartile range of total scores of self‐care (140.00 ± 67), the scores of self‐care maintenance (50 ± 24.76), the scores of self‐care management (56.25 ± 29.41) and the scores of self‐care confidence (54.79 ± 29.17). Age, family model, primary caregiver, maximum systolic blood pressure, coverage of medical insurance, disease duration, receiving self‐care education, education level, economic burden and family history of hypertension were the most powerful predictors of self‐care among older adults with hypertension.

**What does this article contribute to the wider global clinical community?**

1. It was the first time to perform a cross‐sectional survey using the Chinese version of SC‐HI among a large sample of older adults with hypertension in China.
2. The status of self‐care among older adults with hypertension in China is poor.
3. The predictors of self‐care among older adults with hypertension in China are age, education level, economic burden, coverage of medical insurance, family model, primary caregiver, disease duration, maximum systolic blood pressure, family history of hypertension and receiving self‐care education.

## 1 INTRODUCTION

Hypertension is the highest prevalence of cardiovascular diseases in China (Chen et al., 2018). According to the latest report, there are approximately 250 million people with hypertension, with the prevalence rate of 17.9% (Fan et al., 2020). The incidence of hypertension rises as the population grows older (Hansell et al., 2017; Hypertension Branch of Chinese Association for Promotion of International Communication in Medical Care et al., 2019). China has the world's largest elderly population. According to the latest demographic data from the National Bureau of Statistics in 2019, by the end of 2018, China's ageing population aged 60 and above had reached 249 million, accounting for 17.9% of the population (National Bureau of Statistics of China, 2020). Lu et al. (2017) found that over 50% of the older people in China, who have target organ damage, experience hypertension. Therefore, it is very important for medical staff to help elderly patients with hypertension to improve self‐care ability and effectively control their blood pressure.

## 2 BACKGROUND

Hypertension is a chronic lifelong disease. If blood pressure is not well controlled, it can cause multiple organ damage, even disability and death. WHO reported that the cost of chronic diseases, mainly hypertension and its complications, and the loss of labour force have seriously hindered the development of the global economy (Organization, 2002). Every year, China pays over 40 billion yuan in expenses related to hypertension, causing a heavy burden on families and society (Hypertension Branch of Chinese Association for Promotion of International Communication in Medical Care et al., 2019). Nevertheless, the situation of hypertension control in China is not ideal. The insights from the China PEACE Million Persons Project revealed that 86.1% hypertensive patients were untreated and only 10.3% of the untreated were aware of having hypertension among 2,310,184 participants (Mahajan et al., 2019). A study conducted in Jiangsu province showed that the rates of awareness, treatment and control of hypertension were 56.6%, 45.3% and 12.0% (standardized rates: 52.2%, 41.0% and11.2%), respectively, and all the rates were positively associated with age (Su et al., 2019). Despite the methods of preventing and treating hypertension had attracted great attention and support from the government and medical institutions, targeted interventions should be considered taking into account differences in gender, urban and rural areas, age, etc. (Cao et al., 2019; Yan et al., 2020).

The American nursing theorist Dorothy Orem put the self‐care theory forward in 1959. Orem believed that the ultimate goal of nursing is to maximize the maintenance and promotion of self‐care for clients (Orem, 2001). In the self‐care theory, self‐care is a human regulatory function, deliberately engaged in by a person in order to attain structural integrity and human functioning for the purpose of maintaining life, health and well‐being (Orem, 2001). Self‐care refers to the voluntary adjustment activities carried out by individuals to maintain, recover or improve their own health (Tabrizi et al., 2018). It helped patients make remarkable achievements in correcting bad lifestyle, improving treatment compliance, preventing complications, improving quality of life and reducing medical service costs (Chobanian et al., 2003; Dickson et al., 2017; Riegel et al., 2012; Tabrizi et al., 2018; Vaughan et al., 2017). In terms of hypertension, self‐care has been proved to be one of the main determinants of hypertension control (Eckel et al., 2014; Li et al., 2015), which requires patients not only to be treated with standardized medication, but also pay attention to daily blood pressure monitoring, weight control, low‐salt diet and regular exercise, etc. In fact, intervention studies to improve self‐care ability in patients with hypertension have been conducted and reported good results. For example, the use of a tablet computer‐based self‐monitoring system helped to improve blood pressure control (Or & Tao, 2016). Self‐management education tailored to health literacy had been proved to significantly promote patients with hypertension medication adherence (Delavar et al., 2019). Study protocol of clinical trial had published that the effectiveness of a multi‐factorial intervention consists of self‐management of antihypertensive medication, self‐measurement of blood pressure, hypo‐caloric and low‐sodium diet and physical exercise in patients with uncontrolled hypertension taking two or more antihypertensive drugs (Villafuerte et al., 2020). However, lack of hypertension self‐care will often seriously affect patients’ health, especially older adults (Zhao et al., 2019). Haveman‐Nies et al. (2003) reported that the self‐care ability decreases with ageing. Furthermore, Orem (2001) noted that socio‐cultural norms and values affect people, families, communities and self‐care responsibilities. Therefore, it is very important to improve self‐care among older adults with hypertension in China. For formulating effective self‐care interventions, it is necessary to investigate the status and predictors of self‐care among older adults with hypertension in China.

The status of self‐care among patients with hypertension is mainly evaluated by scales (Chen et al., 2014). A literature review showed that several of the following instruments of measuring self‐care among older adults with hypertension are in use (Han, Lee, et al., 2014; Han, Song, et al., 2014). However, they all have room to be improved. The Exercise of Self‐Care Agency scale (ESCA) (Wang & Laffrey, 2000) and the Self‐Care Ability Scale for the Elderly (SASE) (Süderhamn et al., 1996) are generic scales and needs to be more specified in terms of diseases. Hypertension Self‐Care Profile (HBP SCP) (Han, Song, et al., 2014) has too many items, which is not suitable for elderly population. Hypertension Self‐Care Activity Level Effects (H‐SCALE) (Warren‐Findlow et al., 2013; Warren‐Findlow & Seymour, 2011) is also a general and more suitable method for large‐scale epidemiological investigation. Hill‐Bone Compliance to High Blood Pressure Therapy Scale (HBTS) (Kim et al., 2000) only involves the evaluation of taking medicine. Therapeutic Adherence Scale for Hypertensive Patients (TASHP) (Tang et al., 2011) lacks evaluation of symptom management and self‐efficacy.

In 2017, Dickson et al. (2017) developed a 23‐item Self‐Care of Hypertension Inventory (SC‐HI) based on the middle‐range theory of self‐care, which could evaluate the effectiveness of self‐care interventions. In previous study (Zhao et al., 2019), we cross‐culturally adapted SC‐HI into Chinese and it was proved to be a valid and reliable instrument for measuring self‐care among older adults with hypertension in China. In this study, we further used the Chinese version of SC‐HI to investigate the status and the predictors of self‐care among older adults with hypertension. The results of this study will give the theoretical basis and practical reference for further research.

## 3 METHOD

### 3.1 Study design and participants

According to the STROBE statement checklist (for details, see “File S1”), a cross‐sectional observational study was performed. Following the convenient sampling method, we rolled participants of the departments of cardiology and geriatrics of four tertiary hospitals in Nantong City of Jiangsu Province, China from September 2018–February 2019. Based on the sample size of the survey, it is better to estimate 10–20 times of the total items of the main scale (Andreasen et al., 1996). There are 23 items in the Chinese version of SC‐HI and a sample size of 253–552 people is required, considering 10%–20% of the lost follow‐up rate. The inclusion criteria were that patients aged 60 years or older (Xin et al., 2020), being on antihypertensive medications, being able to provide informed consent and communicating without barriers were rolled. Patients were excluded if they had acute or advanced diseases, for example, acute myocardial infarction or advanced cancer, mental illness or other conditions that precluded participation in the study (Zhao et al., 2019). After identification of participants, 544 older adults with hypertension were invited to participate in the study. Before recruiting patients, the institutional review board approved this study.

### 3.2 Data collection

Data were collected in the departments of cardiology and geriatrics in four hospitals (*N* = 544). The researchers explained the purpose and procedure of the study to each participant and participants gave the informed consent. Using paper questionnaires, trained researchers collected data in the one‐on‐one and face‐to‐face interviews. For those who had difficulties in filling out the questionnaires, such as with low education level, degradation of vision and hand shake, the researchers explained the items patiently and helped them with the questionnaires. All the questionnaires were completed on the spot for about an hour a person, with a recovery rate of 100% and no missing entries. Each questionnaire was coded for verification and statistical analysis. All data were typed‐in and checked by two researchers to ensure the accuracy and completeness.

### 3.3 Main research tools

Participants finished the general situation questionnaire and the Chinese version of SC‐HI.

#### 3.3.1 General information questionnaire

After literature review, the general situation questionnaire was compiled containing patients’ demographic data and clinical characteristics, such as gender, age (years), education level, body mass index (BMI, kg/m^2^), marital status, pre‐retirement occupations, the economic burden, family model, primary caregiver, coverage of medical insurance, maximum systolic blood pressure, maximum diastolic blood pressure, stable systolic blood pressure after medication, stable diastolic blood pressure after medication, classification of hypertension, family history of hypertension, disease duration, co‐morbidity (i.e. cardiovascular disease, kidney disease, stroke) and self‐assessment of health. All answers were self‐reported by participants.

#### 3.3.2 Chinese version of SC‐HI

Chinese version of SC‐HI is a self‐rating scale and includes 23 items divided into three subscales: self‐care maintenance, self‐care management and self‐care confidence. Each of the three scales scored separately and standardized from 0–100 with higher scores indicating better self‐care. Self‐care is considered adequate if the separate score is 70 or greater (Silveira et al., 2018). The Chinese version of SC‐HI has the Cronbach's α coefficients of 0.858 (0.690–0.891 for each dimension) and 0.701 (0.662–0.884 for each dimension) for Guttman, and 0.701 (0.676–0.885 for each dimension) for sibue formula (Zhao et al., 2019). The retest reliabilities of self‐care maintenance scale and self‐care confidence scale are 0.975 and 0.996 respectively (*p* < .01) (Zhao et al., 2019). The content validity of the total scale is 0.985, and the item level content validity index is 0.8333–1 (Zhao et al., 2019).

### 3.4 Statistical analysis

Data were analysed using SPSS Version 25.0. Continuous and normally distributed variables were presented as means and standard deviation (mean ± *SD*) and categorical variables as frequencies (%). Variables were used independent sample *t* test of the group difference. Not normally distributed data were described by median and inter‐quartile range (IQR, 25%–75%), and Mann–Whitney *U* test and Kruskal–Wallis *H* were used to assess group differences. We used the univariate generalized liner model correlation regression analysis (GLM) and the optimal scaling regression analysis to investigate the predictors of self‐care of older adults with hypertension. Compared with other analysis methods, the optimal scaling regression analysis has a wider scope of application and the results are more stable and accurate. Statistical significance is considered when *p* < .05 (two tail).

## 4 RESULTS

### 4.1 Patient characteristics

This study included 544 older adults with hypertension aged 60–93 years, with an average age of (70.56 ± 8.75) years old. Among the 544 participants, 286 were males, 258 were females, 466 had a spouse, 502 had medical insurance, 481 lived with their spouse or children, 454 had spouses or children as the primary caregivers, 303 had a BMI above normal, 468 had a disease course of more than 10 years, 66 had hypertension grade 1, 208 had hypertension grade 2 and 270 had hypertension grade 3, 305 had a family history, 333 had complications, 114 had never received health education and 89 had poor self‐reported health (see Table 1 for details).

**TABLE 1:** The demographic and clinical variables of participants

Variables	Frequencies	Percentage (%)
Gender		
Males	286	52.27
Females	258	47.43
Age (years)		
60–74	372	63.38
75–89	158	29.04
≥90	14	2.57
Marital status		
Married	466	85.66
Single/divorced/widowed	78	14.34
Education level (years)		
Primary school and below	234	43.01
Middle school	146	26.84
High school/secondary school	109	20.04
College and above	55	10.11
Pre‐retirement occupations		
Farmers	199	36.58
Workers	150	27.57
Institution staff members	145	26.65
Others	50	9.19
Economic burden		
Light	144	26.47
Average	276	50.74
Heavy	124	22.79
Coverage of medical insurance		
Partial	473	86.95
Full	29	5.33
None	42	7.72
Family model		
Living alone	37	6.80
Living with the spouse	186	34.19
Living with children	68	12.50
Living with the spouse and children	227	4.78
Others	26	
Primary caregiver		
Spouse	283	52.02
Children	171	31.43
Nanny	15	2.76
Others	75	13.79
BMI (kg/m^2^)		
<18.50	26	4.78
18.50–24.99	268	49.26
25.00–29.99	206	37.87
≥30.00	44	8.09
Disease duration (years)		
<1	13	2.39
1–5	153	28.13
>5–10	137	25.18
>10–20	165	30.33
>20–30	47	8.64
>30	29	5.33
Classification of hypertension		
Stage I hypertension	66	12.13
Stage II hypertension	2088	38.24
Stage III hypertension	270	49.63
Maximum systolic blood pressure (mmHg)		
≤159	113	20.77
160–179	204	37.50
180–199	147	27.02
≥200	80	14.71
Maximum diastolic blood pressure (mmHg)		
<90	79	14.52
90–99	148	27.21
100–109	192	35.29
110–119	68	12.50
≥120	57	10.48
Stable systolic blood pressure after medication (mmHg)		
<100	1	0.18
100–119	25	4.60
120–139	318	58.46
140–159	186	34.19
≥160	14	2.57
Stable diastolic blood pressure after medication (mmHg)		
<60	8	1.47
60–79	202	37.13
80–99	322	59.19
100–120	10	1.84
>120	2	0.37
Family history of hypertension		
Yes	305	56.07
No	239	43.93
Co‐morbidity		
0	211	38.79
1	243	44.67
2	58	10.66
≥3	32	5.88
Receiving self‐care education		
Often	189	34.74
Seldom	241	44.30
Never	114	20.96
Self‐assessment of health		
Good	217	39.89
Average	238	43.75
Poor	89	16.36

### 4.2 Self‐care status

The Chinese SC‐HI and three subscales carried out the normality tests of the scores. The results of Kolmogorov–Smirnov tests showed all non‐normally distributed total scores of SC‐HI and the scores of three subscales (shown in Table 2). Table 2 shows the median and inter‐quartile range of the total score of SC‐HI and three subscales’ scores.

**TABLE 2:** Scores of the Chinese version of self‐care of hypertension inventory

Scale	Number of items	Minimum	Maximum	Median	Inter‐quartile range	*Z*	*p*
Self‐care maintenance	11	0	100	50	24.76	0.057	.000**
Self‐care management	6	0	100	56.25	29.41	0.061	.002**
Self‐care confidence	6	0	100	54.79	29.17	0.056	.000**
SC‐HI	23	28.13	300	140.92	67	0.044	.013*

**p < .01; *p < .05.

Based on descriptive statistical analysis, we found that among 544 participants, 477 participants got the total scores <210, suggesting a poor status of self‐care for hypertension. On the subscale of self‐care maintenance, 458 (84.19%) participants got the scores <70, indicating poor self‐care maintenance. In this study, a total of 378 participants who had symptom of elevated blood pressure in the past 1 month filled the subscale of self‐care management. Among them, 278 participants (73.54%) got the scores <70, indicating poor self‐care management. On the subscale of self‐care confidence, 418 (76.84%) participants got the scores <70, indicating poor self‐care confidence.

In the self‐care maintenance domain, the lowest‐score item was item 8 “Ask for low‐salt items when eating out or visiting others?” The highest‐score item was item 7 “Take medicines as prescribed?” In the self‐care management domain, the lowest‐score item was item 12 “How quickly did you recognize that your blood pressure was up?” The highest‐score item was item 15 “Be careful to take your prescription medicines more regularly?” In the self‐care confidence domain, the lowest‐score item was item 21 “Evaluate changes in your blood pressure?” The highest‐score item was item 19 “Follow your treatment regimen?” (see Table 3 for details).

**TABLE 3:** Scores of each item in the Chinese version of Self‐Care of Hypertension Inventory

	Item	Median	Inter‐quartile range	Average	Standard deviation	Ranking
Self‐care maintenance	1	3	1	2.65	0.89	6
	2	3	2	3.03	0.77	2
	3	3	2	2.93	0.97	3
	4	3	1	2.46	1.03	9
	5	3	2	2.80	0.91	4
	6	3	2	2.61	1.13	7
	7	4	1	3.47	0.75	1
	8	2	1	1.78	0.92	11
	9	2	2	2.58	1.07	8
	10	3	1	2.77	0.85	5
	11	2	2	1.91	1.00	10
Self‐care management	12	2	1	1.69	1.17	6
	13	3	2	2.91	0.92	3
	14	3	2	2.91	0.84	3
	15	4	1	3.51	0.69	1
	16	3	1	3.06	0.81	2
	17	3	1	2.51	1.02	5
Self‐care confidence	18	3	1	2.82	0.72	4
	19	3	1	3.12	0.70	1
	20	3	1	2.70	0.76	5
	21	3	1	2.67	0.79	6
	22	3	1	2.88	0.73	2
	23	3	1	2.84	0.78	3

### 4.3 GLM univariate analysis of predictors of self‐care

We performed GLM univariate analysis to preliminarily analyse the predictors of self‐care. The demographic and clinical data of participants were the independent variables and the scores of self‐care maintenance scale, self‐care management scale, self‐care confidence scale and SC‐HI were the dependent variables. According to the requirements of the variables in the analysis, the specific assignments of variables are shown in Table 4.

**TABLE 4:** Assignment of variables

Variables name	Variables	Assigning method
Gender	X_1_	X_1_ = Males, X_2_ = Females
Age (years)	X_2_	X_1_ = 60–74, X_2_ = 75–89, X_3_ ≥ 90
Marital status	X_3_	X_1_ = Married, X_2_ = Single/divorced/widowed
Education level (years)	X_4_	X_1_ = Primary school or below, X_2_ = Middle school, X_3_ = High school/secondary school, X_4_ = College degree or above
Pre‐retirement occupations	X_5_	X_1_ = Framers, X_2_ = Workers, X_3_ = Institution staff members, X_4_ = Others
Economic burden	X_6_	X_1_ = Light, X_2_ = Average, X_3_ = Heavy
Coverage of medical insurance	X_7_	X_1_ = Partial, X_2_ = Full, X_3_ = None
Family model	X_8_	X_1_ = Living alone, X_2_ = Living with the spouse, X_3_ = Living with children, X_4_ = Living with the spouse and children, X_5_ = Others
Primary caregiver	X_9_	X_1_ = Spouse, X_2_ = Children, X_3_ = Nanny, X_4_ = Others
BMI (kg/m^2^)	X_10_	X_1_ < 18.50, X_2_ = 18.50–24.99, X_3_ = 25.00–29.99, X_4_ ≥ 30.00
Disease duration (years)	X_11_	X_1_ < 1, X_2_ = 1–5, X_3_ > 5–10, X_4_ > 10–20, X_5_ > 20–30, X_6_ > 30
Classification of hypertension	X_12_	X_1_ = Stage I hypertension, X_2_ = Stage II hypertension, X_3_ = Stage III hypertension
Maximum systolic blood pressure (mmHg)	X_13_	X_1_ ≤ 159, X_2_ = 160–179, X_3_ = 180–199, X_4_ ≥ 200
Maximum diastolic blood pressure (mmHg)	X_14_	X_1_ < 90, X_2_ = 90–99, X_3_ = 100–109, X_4_ = 110–119, X5 ≥ 120
Stable systolic blood pressure after medication (mmHg)	X_15_	X_1_ < 100, X_2_ = 100–119, X_3_ = 120–139, X_4_ = 140–159, X_5_ ≥ 160
Stable diastolic blood pressure after medication (mmHg)	X_16_	X_1_ < 60, X_2_ = 60–79, X_3_ = 80–99, X_4_ = 100–120, X_5_ > 120
Family history of hypertension	X_17_	X_1_ = Yes, X_2_ = No
Co‐morbidity	X_18_	X_1_ = 0, X_2_ = 1, X_3_ = 2, X_4_ ≥ 3
Receiving self‐care education	X_19_	X_1_ = Often, X_2_ = Seldom, X_3_ = Never
Self‐assessment of health	X_20_	X_1_ = Good, X_2_ = Average, X_3_ = Poor

#### 4.3.1 GLM univariate analysis of predictors of self‐care maintenance

The results showed that participants being married, being institution staff members, having light or average economic burden, living with the spouse, having the maximum diastolic blood pressure of 90–99, having the stable diastolic blood pressure of 60–79, 80–99 and 100–120, having family history of hypertension, often receiving self‐care education and their primary caregivers being spouse or children, the self‐assessment of health being good had the statistically significant difference comparing with control group (*p* < .05), as shown in Table 5.

**TABLE 5:** GLM univariate analysis of predictors of self‐care maintenance

Variables	B	Wald	*p*	95% CI	
				Upper bound	Lower bound
Gender (referring to females)	−0.286	2.911	.088	−0.615	0.043
Age (years) (referring to ≥90)					
60–74	0.384	0.490	.484	−0.692	1.461
75–89	0.271	0.249	.617	−0.792	1.334
Marital status (referring to single/divorced/widowed)	−0.741	5.168	.023*	−1.380	−0.102
Education level (years) (referring to college degree or above)					
Primary school or below	−0.079	0.049	.825	−0.778	0.621
Middle school	0.216	0.431	.511	−0.429	0.860
High school/secondary school	0.100	0.104	.747	−0.506	0.706
Pre‐retirement occupations (referring to others)					
Farmers	0.168	0.280	.597	−0.455	0.792
Workers	0.392	1.665	.197	−0.204	0.988
Institution staff members	1.157	11.834	.001**	0.498	1.817
Economic burden (referring to heavy)					
Light	−0.935	11.985	.001**	−1.464	−0.406
Average	−0.538	5.619	.018*	−0.982	−0.093
Coverage of medical insurance (referring to none)					
Partial	0.166	0.286	.593	−0.442	0.773
Full	−0.187	0.143	.705	−1.156	0.782
Family model (referring to others)					
Living alone	−0.088	0.031	.861	−1.067	0.891
Living with the spouse	1.051	5.586	.018*	0.179	1.922
Living with children	0.217	0.222	.638	−0.687	1.122
Living with the spouse and children	0.561	1.712	.191	−0.280	1.402
Primary caregiver (referring to others)					
Spouse	−0.651	5.895	.015*	−1.176	−0.125
Children	−0.848	9.404	.002**	−1.390	−0.306
Nanny	0.337	0.352	.553	−0.775	1.448
BMI (kg/m^2^) (referring to ≥30.00)					
<18.50	0.186	0.171	.679	−0.698	1.071
18.50–24.99	0.210	0.470	.493	−0.391	0.811
25.00–29.99	0.479	2.499	.114	−0.115	1.072
Disease duration (years) (referring to >30)					
<1	−0.851	1.830	.176	−2.083	0.382
1–5	−0.157	0.152	.696	−0.945	0.631
>5–10	0.111	0.077	.782	−0.676	0.898
>10–20	−0.392	1.004	.316	−1.158	0.375
>20–30	0.354	0.634	.426	−0.517	1.224
Classification of hypertension (referring to stage III hypertension)					
Stage I hypertension	0.396	1.304	.253	−0.283	1.075
Stage II hypertension	−0.019	0.007	.933	−0.471	0.432
Maximum systolic blood pressure (mmHg) (referring to ≥200)					
≤159	−0.159	0.193	.661	−0.866	0.549
160–179	−0.123	0.173	.678	−0.702	0.456
180–199	−0.216	0.628	.428	−0.751	0.318
Maximum diastolic blood pressure (mmHg) (referring to ≥120)					
<90	−0.590	2.531	.112	−1.317	0.137
90–99	−0.704	4.828	.028*	−1.332	−0.076
100–109	−0.264	0.758	.384	−0.859	0.330
110–119	−0.308	0.791	.374	−0.988	0.371
Stable systolic blood pressure after medication (mmHg) (referring to ≥160)					
<100	−0.264	0.017	.896	−4.234	3.705
100–119	0.297	0.203	.652	−0.996	1.591
120–139	0.072	0.017	.895	−0.997	1.142
140–159	0.216	0.160	.689	−0.843	1.275
Stable diastolic blood pressure after medication (mmHg) (referring to >120)					
<60	1.547	1.330	.249	−1.082	4.175
60–79	2.435	4.682	.030*	0.229	4.640
80–99	2.231	4.000	.046*	0.045	4.417
100–120	2.998	5.897	.015*	0.578	5.417
Family history of hypertension (referring to no)	0.324	3.946	.047*	0.004	0.644
Co‐morbidity (referring to ≥3)					
0	−0.071	0.037	.847	−0.792	0.650
1	0.300	0.693	.405	−0.406	1.005
2	0.200	0.234	.628	−0.610	1.009
Receiving self‐care education (referring to never)					
Often	0.895	12.933	.000**	0.407	1.382
Seldom	0.000	0.000	1.000	−0.433	0.432
Self‐assessment of health (referring to poor)					
Good	0.579	4.183	.041*	0.024	1.135
Average	0.340	1.818	.178	−0.154	0.833

**p < .01; *p < .05.

#### 4.3.2 GLM univariate analysis of predictors of self‐care management

The results showed that participants having light or average economic burden, having the maximum systolic blood pressure of ≤159, 160–179 and 180–199 and their primary caregivers being spouse had the statistically significant difference comparing with control group (*p* < .05), as shown in Table 6.

**TABLE 6:** GLM univariate analysis of predictors of self‐care management

Variables	B	Wald	*p*	95% CI	
				Upper bound	Lower bound
Gender (referring to females)	−0.136	0.441	.506	−0.538	0.266
Age (years) (referring to ≥90)					
60–74	0.649	0.922	.337	−0.676	1.975
75–89	−0.103	0.024	.877	−1.410	1.204
Marital status (referring to single/divorced/widowed)	−0.297	0.568	.451	−1.068	0.475
Education level (years) (referring to college degree or above)					
Primary school or below	0.341	0.677	.411	−0.471	1.153
Middle school	0.209	0.293	.588	−0.547	0.965
High school/secondary school	0.234	0.379	.538	−0.511	0.979
Pre‐retirement occupations (referring to others)					
Farmers	−0.216	0.344	.558	−0.938	0.506
Workers	−0.133	0.147	.702	−0.816	0.549
Institution staff members	0.692	3.388	.066	−0.045	1.428
Economic burden (referring to heavy)					
Light	−0.575	2.752	.097	−1.255	0.104
Average	−0.855	7.740	.005**	−1.457	−0.253
Coverage of medical insurance (referring to none)					
Partial	0.514	1.558	.212	−0.293	1.320
Full	−0.009	0.000	.989	−1.233	1.216
Family model (referring to others)					
Living alone	1.103	3.239	.072	−0.098	2.305
Living with the spouse	0.916	2.755	.097	−0.166	1.997
Living with children	0.569	1.001	.317	−0.545	1.682
Living with the spouse and children	0.623	1.375	.241	−0.418	1.665
Primary caregiver (referring to others)					
Spouse	−0.700	4.998	.025*	−1.314	−0.086
Children	−0.736	5.246	.022*	−1.365	−0.106
Nanny	0.525	0.542	.461	−0.873	1.923
BMI (kg/m^2^) (referring to ≥30.00)					
<18.50	−0.076	0.020	.887	−1.129	0.977
18.50–24.99	0.247	0.401	.527	−0.517	1.010
25.00–29.99	0.487	1.529	.216	−0.285	1.260
Disease duration (years) (referring to >30)					
<1	0.026	0.001	.970	−1.298	1.350
1–5	−0.520	1.284	.257	−1.420	0.379
>5–10	−0.136	0.087	.768	−1.039	0.768
>10–20	−0.140	0.100	.751	−1.009	0.728
>20–30	0.446	0.730	.393	−0.577	1.469
Classification of hypertension (referring to stage III hypertension)					
Stage I hypertension	0.313	0.547	.460	−0.516	1.142
Stage II hypertension	0.047	0.025	.874	−0.528	0.621
Maximum systolic blood pressure (mmHg) (referring to ≥200)					
≤159	−1.231	7.253	.007**	−2.126	−0.335
160–179	−0.984	7.366	.007**	−1.695	−0.273
180–199	−0.677	4.687	.030*	−1.289	−0.064
Maximum diastolic blood pressure (mmHg) (referring to ≥120)					
<90	−0.284	0.397	.529	−1.166	0.598
90–99	−0.072	0.034	.854	−0.838	0.694
100–109	−0.105	0.079	.779	−0.837	0.627
110–119	0.093	0.049	.824	−0.731	0.917
Stable systolic blood pressure after medication (mmHg) (referring to ≥160)					
<100	−2.966	1.887	.170	−7.198	1.266
100–119	0.103	0.020	.889	−1.339	1.545
120–139	−0.145	0.060	.806	−1.299	1.009
140–159	−0.427	0.532	.466	−1.574	0.720
Stable diastolic blood pressure after medication (mmHg) (referring to >120)					
<60	−0.066	0.002	.964	−2.951	2.819
60–79	0.456	0.153	.695	−1.827	2.740
80–99	−0.043	0.001	.970	−2.301	2.215
100–120	−0.101	0.006	.938	−2.634	2.432
Family history of hypertension (referring to no)	0.067	0.114	.736	−0.321	0.454
Co‐morbidity (referring to ≥3)					
0	0.114	0.059	.808	−0.804	1.033
1	0.475	1.018	.313	−0.448	1.397
2	0.475	0.783	.376	−0.577	1.527
Receiving self‐care education (referring to never)					
Often	0.454	2.253	.133	−0.139	1.046
Seldom	−0.345	1.684	.194	−0.867	0.176
Self‐assessment of health (referring to poor)					
Good	0.281	0.559	.455	−0.455	1.016
Average	−0.040	0.014	.905	−0.706	0.625

**p < .01; *p < .05.

#### 4.3.3 GLM univariate analysis of predictors of self‐care confidence

The results showed that participants having 0 or 1 co‐morbidity, using partial, and their self‐assessment of health being average had the statistically significant difference comparing with control group (*p* < .05), as shown in Table 7.

**TABLE 7:** GLM univariate analysis of predictors of self‐care confidence

Variables	B	Wald	*p*	95% CI	
				Upper bound	Lower bound
Gender (referring to females)	0.191	1.290	.256	−0.138	0.520
Age (years) (referring to ≥90)					
60–74	−0.056	0.010	.919	−1.135	1.023
75–89	−0.569	1.096	.295	−1.635	0.496
Marital status (referring to single/divorced/widowed)	0.099	0.093	.760	−0.538	0.737
Education level (years) (referring to college degree or above)					
Primary school or below	−0.350	0.955	.328	−1.051	0.352
Middle school	−0.175	0.283	.595	−0.821	0.470
High school/secondary school	−0.089	0.082	.775	−0.696	0.519
Pre‐retirement occupations (referring to others)					
Farmers	0.294	0.852	.356	−0.330	0.919
Workers	0.140	0.213	.644	−0.456	0.736
Institution staff members	0.603	3.238	.072	−0.054	1.260
Economic burden (referring to heavy)					
Light	0.183	0.467	.495	−0.343	0.709
Average	−0.153	0.456	.500	−0.597	0.291
Coverage of medical insurance (referring to none)					
Partial	−0.811	6.750	.009**	−1.423	−0.199
Full	−0.770	2.402	.121	−1.744	0.204
Family model (referring to others)					
Living alone	0.073	0.021	.884	−0.907	1.054
Living with the spouse	−0.151	0.116	.734	−1.021	0.718
Living with children	−0.183	0.156	.693	−1.089	0.723
Living with the spouse and children	−0.092	0.046	.830	−0.933	0.749
Primary caregiver (referring to others)					
Spouse	−0.333	1.550	.213	−0.858	0.191
Children	−0.497	3.254	.071	−1.038	0.043
Nanny	−0.956	2.820	.093	−2.071	0.160
BMI (kg/m^2^) (referring to ≥30.00)					
<18.50	0.136	0.090	.764	−0.750	1.021
18.50–24.99	−0.072	0.055	.815	−0.673	0.530
25.00–29.99	0.424	1.957	.162	−0.170	1.019
Disease duration (years) (referring to >30)					
<1	−0.762	1.462	.227	−1.996	0.473
1–5	−0.345	0.734	.392	−1.135	0.445
>5–10	−0.349	0.753	.385	−1.138	0.440
>10–20	−0.088	0.051	.821	−0.856	0.679
>20–30	0.237	0.284	.594	−0.635	1.109
Classification of hypertension (referring to stage III hypertension)					
Stage I hypertension	0.022	0.004	.949	−0.658	0.702
Stage II hypertension	−0.347	2.260	.133	−0.800	0.106
Maximum systolic blood pressure (mmHg) (referring to ≥200)					
≤159	−0.158	0.192	.662	−0.868	0.551
160–179	−0.037	0.016	.900	−0.617	0.543
180–199	−0.223	0.665	.415	−0.758	0.313
Maximum diastolic blood pressure (mmHg) (referring to ≥120)					
<90	0.127	0.117	.733	−0.600	0.854
90–99	−0.126	0.156	.693	−0.754	0.501
100–109	−0.202	0.440	.507	−0.797	0.394
110–119	−0.423	1.483	.223	−1.104	0.258
Stable systolic blood pressure after medication (mmHg) (referring to ≥160)					
<100	−3.691	3.156	.076	−7.762	0.381
100–119	0.416	0.396	.529	−0.880	1.712
120–139	0.298	0.298	.585	−0.773	1.370
140–159	0.041	0.006	.940	−1.020	1.102
Stable diastolic blood pressure after medication (mmHg) (referring to >120)					
<60	2.296	2.917	.088	−0.339	4.932
60–79	1.633	2.109	.146	−0.571	3.838
80–99	1.589	2.029	.154	−0.597	3.775
100–120	−0.226	0.034	.854	−2.641	2.189
Family history of hypertension (referring to no)	0.108	0.437	.509	−0.212	0.427
Co‐morbidity (referring to ≥3)					
0	0.810	4.802	.028*	0.086	1.535
1	1.086	8.971	.003**	0.375	1.796
2	0.329	0.632	.427	−0.482	1.140
Receiving self‐care education (referring to never)					
Often	0.139	0.319	.572	−0.345	0.624
Seldom	−0.411	3.441	.064	−0.845	0.023
Self‐assessment of health (referring to poor)					
Good	0.260	0.840	.359	−0.296	0.815
Average	−0.533	4.441	.035*	−1.028	−0.037

**p < .01; *p < .05.

#### 4.3.4 GLM univariate analysis of predictors of self‐care

The results showed that participants being institution staff members, living with the spouse, having the maximum systolic blood pressure of ≤159, 160–179 and 180–199, having 0 or 1 co‐morbidity and their primary caregivers being spouse or children, the self‐assessment of health being good had the statistically significant difference comparing with control group (*p* < .05), as shown in Table 8.

**TABLE 8:** GLM univariate analysis of predictors of self‐care

Variables	B	Wald	*p*	95% CI	
				Upper bound	Lower bound
Gender (referring to females)	−0.009	0.003	.958	−0.337	0.319
Age (years) (referring to ≥90)					
60–74	0.543	0.978	.323	−0.533	1.619
75–89	−0.036	0.004	.947	−1.098	1.026
Marital status (referring to single/divorced/widowed)	−0.300	0.852	.356	−0.936	0.337
Education level (years) (referring to college degree or above)					
Primary school or below	0.137	0.149	.700	−0.561	0.836
Middle school	0.298	0.823	.364	−0.346	0.942
High school/secondary school	0.355	1.318	.251	−0.251	0.961
Pre‐retirement occupations (referring to others)					
Farmers	−0.177	0.311	.577	−0.800	0.446
Workers	−0.046	0.023	.880	−0.640	0.549
Institution staff members	0.680	4.140	.042*	0.025	1.336
Economic burden (referring to heavy)					
Light	0.098	0.135	.714	−0.426	0.623
Average	−0.104	0.213	.645	−0.547	0.338
Coverage of medical insurance (referring to none)					
Partial	0.015	0.002	.962	−0.592	0.622
Full	−0.475	0.922	.337	−1.444	0.494
Family model (referring to others)					
Living alone	0.674	1.820	.177	−0.305	1.653
Living with the spouse	0.952	4.599	.032*	0.082	1.822
Living with children	0.654	2.009	.156	−0.251	1.559
Living with the spouse and children	0.485	1.283	.257	−0.354	1.325
Primary caregiver (referring to others)					
Spouse	−1.028	14.538	.000**	−1.556	−0.499
Children	−1.138	16.787	.000**	−1.682	−0.594
Nanny	−0.174	0.094	.759	−1.284	0.936
BMI (kg/m^2^) (referring to ≥30.00)					
<18.50	0.398	0.780	.377	−0.486	1.282
18.50–24.99	0.249	0.661	.416	−0.351	0.849
25.00–29.99	0.557	3.387	.066	−0.036	1.151
Disease duration (years) (referring to >30)					
<1	−0.116	0.034	.854	−1.346	1.115
1–5	−0.547	1.848	.174	−1.335	0.242
>5–10	−0.225	0.314	.575	−1.011	0.562
>10–20	−0.334	0.733	.392	−1.100	0.431
>20–30	−0.055	0.016	.901	−0.925	0.814
Classification of hypertension (referring to Stage III hypertension)					
Stage I hypertension	0.322	0.864	.353	−0.357	1.000
Stage II hypertension	−0.119	0.267	.606	−0.570	0.332
Maximum systolic blood pressure (mmHg) (referring to ≥200)					
≤159	−1.277	12.315	.000**	−1.991	−0.564
160–179	−0.826	7.745	.005**	−1.408	−0.244
180–199	−0.626	5.241	.022*	−1.162	−0.090
Maximum diastolic blood pressure (mmHg) (referring to ≥120)					
<90	0.184	0.246	.620	−0.542	0.909
90–99	0.055	0.030	.863	−0.570	0.680
100–109	−0.058	0.036	.849	−0.651	0.536
110–119	−0.076	0.048	.826	−0.755	0.602
Stable systolic blood pressure after medication (mmHg) (referring to ≥160)					
<100	−2.082	1.057	.304	−6.051	1.887
100–119	−0.029	0.002	.966	−1.321	1.264
120–139	−0.273	0.251	.616	−1.342	0.796
140–159	−0.479	0.786	.375	−1.537	0.580
Stable diastolic blood pressure after medication (mmHg) (referring to >120)					
<60	0.906	0.458	.498	−1.717	3.528
60–79	1.146	1.047	.306	−1.049	3.341
80–99	0.955	0.740	.390	−1.221	3.131
100–120	0.750	0.374	.541	−1.655	3.156
Family history of hypertension (referring to no)	0.215	1.752	.186	−0.104	0.535
Co‐morbidity (referring to ≥3)					
0	0.766	4.316	.038*	0.043	1.489
1	0.880	5.942	.015*	0.172	1.587
2	0.463	1.258	.262	−0.346	1.272
Receiving self‐care education (referring to never)					
Often	0.473	3.663	.056	−0.011	0.957
Seldom	−0.324	2.148	.143	−0.756	0.109
Self‐assessment of health (referring to poor)					
Good	0.681	5.775	.016*	0.126	1.237
Average	0.204	0.658	.417	−0.289	0.697

**p < .01; *p < .05.

### 4.4 The optimal scaling regression analysis of predictors of self‐care

Although we had the results of the GLM univariate analyses, we still could not discriminate the combined effects and gradient effects of various factors on self‐care, self‐care maintenance, self‐care management and self‐care confidence. Therefore, we used the optimal scaling regression analysis for further analysing predictors. The results of the optimal scaling regression analysis showed that the regression models were statistically significant. However, the multiple correlation coefficient, coefficient of determination and adjusted coefficient of determination of the models were not very ideal, indicating that there may be other predictors that had not yet been included in the equation and should be investigated in future research.

#### 4.4.1 The predictors of self‐care maintenance

The results of the optimal scaling regression analysis showed that the coverage of medical insurance and disease duration were the important predictors of self‐care maintenance (*p* < .05). By combining the partial regression coefficients and the meaning of assignments of original variables, it could be known that participants having long disease duration and no medical insurance had better self‐care maintenance, as shown in Table 9.

**TABLE 9:** The results of partial regression analysis and significance test of predictors of self‐care maintenance

Variables	B	*SE*	*t*	*p*
Gender	−2.947	2.068	−1.425	.155
Age (years)	−3.921	2.114	−1.854	.064
Marital status	−1.616	3.088	−0.523	.601
Education level (years)	1.765	1.244	1.418	.157
Pre‐retirement occupations	−1.314	1.224	−1.073	.284
Economic burden	−2.540	1.638	−1.551	.122
Coverage of medical insurance	4.560	1.764	2.584	.010*
Family model	−0.531	0.952	−0.558	.577
Primary caregiver	0.060	1.074	0.056	.956
BMI (kg/m^2^)	2.275	1.403	1.622	.105
Disease duration (years)	1.825	0.890	2.051	.041*
Classification of hypertension	0.515	2.024	0.255	.799
Maximum systolic blood pressure (mmHg)	1.267	1.412	0.898	.370
Maximum diastolic blood pressure (mmHg)	−0.548	1.012	−0.541	.589
Stable systolic blood pressure after medication (mmHg)	−3.124	1.789	−1.746	.081
Stable diastolic blood pressure after medication (mmHg)	−1.945	2.065	−0.942	.347
Family history of hypertension	−0.488	1.992	−0.245	.807
Co‐morbidity	−1.212	1.264	−0.959	.338
Receiving self‐care education	−2.760	1.524	−1.811	.071
Self‐assessment of health	−1.594	1.681	−0.948	.343

**p < .01; *p < .05.

#### 4.4.2 The predictors of self‐care management

The results of the optimal scaling regression analysis showed that age, maximum systolic blood pressure and receiving self‐care education were the significant predictors of self‐care management (*p* < .05). By combining the partial regression coefficients and the meaning of assignments of original variables, it could be known that participants would have better self‐care management if they were younger, often received self‐care education and their maximum systolic blood pressure were higher, as shown in Table 10.

**TABLE 10:** The results of partial regression analysis and significance test of predictors of self‐care management

Variables	B	*SE*	*t*	*p*
Gender	−0.382	2.323	−0.164	.870
Age (years)	−4.906	2.377	−2.065	.040*
Marital status	0.606	3.515	0.173	.863
Education level (years)	−0.228	1.362	−0.167	.867
Pre‐retirement occupations	0.918	1.312	0.699	.485
Economic burden	0.404	1.886	0.214	.831
Coverage of medical insurance	−2.213	2.108	−1.050	.295
Family model	−1.840	1.079	−1.705	.089
Primary caregiver	2.187	1.162	1.882	.061
BMI (kg/m^2^)	1.742	1.583	1.101	.272
Disease duration (years)	1.690	0.986	1.714	.087
Classification of hypertension	−2.443	2.276	−1.073	.284
Maximum systolic blood pressure (mmHg)	5.234	1.652	3.169	.002**
Maximum diastolic blood pressure (mmHg)	0.658	1.113	0.591	.555
Stable systolic blood pressure after medication (mmHg)	−1.235	1.969	−0.628	.531
Stable diastolic blood pressure after medication (mmHg)	−3.380	2.261	−1.495	.136
Family history of hypertension	−1.028	2.247	−0.458	.648
Co‐morbidity	1.000	1.419	0.705	.481
Receiving self‐care education	−4.418	1.711	−2.582	.010*
Self‐assessment of health	0.011	1.975	0.005	.996

**p < .01; *p < .05.

#### 4.4.3 The predictors of self‐care confidence

The results of the optimal scaling regression analysis showed that education level, economic burden, family model, primary caregiver, family history of hypertension and receiving self‐care education were the significant predictors of self‐care confidence (*p* < .05). By combining the partial regression coefficients and the meaning of assignments of original variables, it could be known that participants would have better self‐care confidence if they had higher education level, had heavier economic burden, lived alone, had other primary caregivers, had family history of hypertension and often received self‐care education, as shown in Table 11.

**TABLE 11:** The results of partial regression analysis and significance test of predictors of self‐care confidence

Variables	B	*SE*	*t*	*p*
Gender	1.797	1.588	1.132	.258
Age (years)	−0.850	1.624	−0.524	.601
Marital status	−0.165	2.372	−0.069	.945
Education level (years)	2.215	0.956	2.318	.021*
Pre‐retirement occupations	0.981	0.940	1.043	.297
Economic burden	3.381	1.258	2.688	.007**
Coverage of medical insurance	−0.882	1.355	−0.651	.515
Family model	−1.710	0.731	−2.341	.020*
Primary caregiver	1.781	0.825	2.158	.031*
BMI (kg/m^2^)	0.953	1.077	0.885	.377
Disease duration (years)	0.715	0.683	1.047	.296
Classification of hypertension	−0.270	1.554	−0.174	.862
Maximum systolic blood pressure (mmHg)	−0.353	1.084	−0.326	.745
Maximum diastolic blood pressure (mmHg)	1.034	0.777	1.330	.184
Stable systolic blood pressure after medication (mmHg)	0.916	1.374	0.667	.505
Stable diastolic blood pressure after medication (mmHg)	−1.327	1.586	−0.837	.403
Family history of hypertension	−3.016	1.530	−1.972	.049*
Co‐morbidity	1.313	0.970	1.353	.177
Receiving self‐care education	−5.288	1.170	−4.518	.000**
Self‐assessment of health	−1.866	1.291	−1.445	.149

**p < .01; *p < .05.

#### 4.4.4 The predictors of self‐care

The results of the optimal scaling regression analysis showed that age, family model, primary caregiver, maximum systolic blood pressure and receiving self‐care education were the significant predictors of self‐care (*p* < .05). By combining the partial regression coefficients and the meaning of assignments of original variables, it could be known that participants would have better self‐care if they were younger, lived alone, had other primary caregivers, often received self‐care education and their maximum systolic blood pressure were higher, as shown in Table 12.

**TABLE 12:** The results of partial regression analysis and significance test of predictors of self‐care

Variables	B	*SE*	*t*	*p*
Gender	−1.240	4.444	−0.279	.780
Age (years)	−10.695	4.544	−2.354	.019*
Marital status	−4.300	6.637	−0.648	.517
Education level (years)	2.773	2.674	1.037	.300
Pre‐retirement occupations	4.187	2.631	1.592	.112
Economic burden	−3.018	3.520	−0.857	.392
Coverage of medical insurance	−0.603	3.792	−0.159	.874
Family model	−5.837	2.045	−2.854	.004**
Primary caregiver	7.073	2.309	3.063	.002**
BMI (kg/m^2^)	2.401	3.014	0.797	.426
Disease duration (years)	2.868	1.912	1.500	.134
Classification of hypertension	−2.383	4.349	−0.548	.584
Maximum systolic blood pressure (mmHg)	11.124	3.034	3.666	.000**
Maximum diastolic blood pressure (mmHg)	−1.056	2.176	−0.486	.627
Stable systolic blood pressure after medication (mmHg)	−3.212	3.846	−0.835	.404
Stable diastolic blood pressure after medication (mmHg)	−6.464	4.439	−1.456	.146
Family history of hypertension	−4.208	4.281	−0.983	.326
Co‐morbidity	−3.819	2.715	−1.406	.160
Receiving self‐care education	−10.176	3.276	−3.107	.002**
Self‐assessment of health	−5.954	3.613	−1.648	.100

**p < .01; *p < .05.

#### 4.4.5 Summary of related predictors

This study indicated that age, family model, primary caregiver, maximum systolic blood pressure, receiving self‐care education are the predictors of self‐care. Participants would have better self‐care if they were younger, lived alone, had other primary caregivers, often received self‐care education and had higher maximum systolic blood pressure. Coverage of medical insurance and disease duration are the predictors of self‐care maintenance. They would have better self‐care maintenance if they had none of medical insurance and long disease duration. Age, high systolic blood pressure and receiving self‐care education are the predictors of self‐care management. They would have better self‐care management if they were younger, had higher maximum systolic blood pressure and often received self‐care education. Education level, economic burden, family model, primary caregiver, family history of hypertension and receiving self‐care education are the predictors of self‐care confidence. They would have better self‐care confidence if they had higher education level, had heavier economic burden, lived alone, had other primary caregivers and had family history of hypertension and often received self‐care education. In order to compare all the predictors of self‐care, self‐care maintenance, self‐care management and self‐care confidence, we summarized all the predictors in one table, as shown in Table 13.

**TABLE 13:** Summary of predictors of SC‐HI and three domains

Variables	SC‐HI	Self‐care maintenance	Self‐care management	Self‐care confidence
Age (years)	+		+	
Education level (years)				+
Economic burden				+
Coverage of medical insurance		+		
Family model	+			+
Primary caregiver	+			+
Disease duration (years)		+		
Maximum systolic blood pressure (mmHg)	+		+	
Family history of hypertension				+
Receiving self‐care education	+		+	+

## 5 DISCUSSION

### 5.1 Overall evaluation of self‐care status

According to Orem's opinion, the ultimate goal of nursing is to maximize the maintenance and promotion of self‐care for clients (Orem, 2001). In order to promote the self‐care ability of elderly patients with hypertension in China, a cross‐sectional study was performed to investigate the status of self‐care among 544 elderly patients with hypertension in China using the Chinese version of SC‐HI. The median and inter‐quartile range of total scores of self‐care was 140.00 ± 67 that of total scores of self‐care maintenance was 50 ± 24.76, that of total scores of self‐care management was 56.25 ± 29.41 and that of total scores of self‐care confidence was 54.79 ± 29.17. According to the classification criteria of the original scale, the self‐care status of patients is good when the score of each dimension is >70 (Silveira et al., 2018). Therefore, according to the scale, the results of this study showed that the status of self‐care among older adults with hypertension in China is poor. This result is consistent with Ademe et al.’s (2019) research, which indicates that self‐care of older adults with hypertension needs to be improved urgently. In this study, the score of self‐care maintenance was the lowest, which indicates that elderly patients lack awareness of long‐term disease management. The medical staff should cultivate older adults with hypertension knowledge of long‐term disease and belief of lifelong self‐care. Self‐care confidence refers to the patient's confidence in controlling symptoms and treatment compliance (Riegel & Dickson, 2008). In this study, participants got the highest scores in this dimension. The reasons might be that the participants were all inpatients who could receive good professional support and felt safe enough.

### 5.2 Analysis of the self‐care maintenance status

In the subscale of self‐care maintenance, participants got the highest scores of item 7 “Take medicines as prescribed?” Of participants, 331 (60.85%) were able to take medicines daily, and it indicates good medication adherence, which was consistent with Ma et al.’s (2019) research. The possible reason is that with the popularization of health education, most elderly patients have realized the importance of taking medicine during the hypertension treatment and are able to stick to doing it. The lowest scoring item was item 8 “Ask for low salt items when eating out or visiting others?” Of participants, 267 (49.08%) answered “never or rarely.” About item 5 “Eat a low‐salt diet”, 208 (38.42%) participants could not guarantee the daily salt intake <6 g, which was consistent with Li et al.’s (2014) research. The reasons might be related to Chinese food culture and individuals’ eating habits. The food in Chinese restaurant often has heavy taste. Some of elderly people like eating pickled food.

### 5.3 Analysis of the self‐care management status

In this study, 378 participants had elevated blood pressure in the past 1 month. The highest scoring item was “Be careful to take your prescription medicines more regularly”. Of participants, 339 (89.68%) answered that they would take antihypertensive drugs more regularly, while only 2 (0.53%) participants would not take antihypertensive drugs, which was consistent with Ma et al.’s (2019) research. Taking anti‐hypertensive drugs as prescribed by doctors is the most important measure to control hypertension, stabilize the range of blood pressure fluctuations and reduce the incidence of cardiovascular diseases. In recent years, almost all antihypertensive drugs had been included into the scope of medical insurance, which greatly reduced the financial burden of patients and improved medication adherence. In this study, only 91 (16.73%) elderly patients were always or daily able to measure their blood pressure, which was lower than that reported by Fu‐wai Hospital Chinese Academy of Medical Sciences (Lu et al., 2017). The possible reason is that the participants in the study were all older adults, who were far less familiar with sphygmomanometers than younger patients. Therefore, the low self‐test rate, improper use of the blood pressure meter and inaccurate measurement would lead to miss recognizing fluctuations of blood pressure. In the item of “Evaluate how well an action works?,” 19 (5.03%) participants did not take any measures to deal with elevated blood pressure and others were certain or very certain that the measures taken would reduce blood pressure effectively. It indicates that there is still some room for further improvement.

### 5.4 Analysis of the self‐care confidence status

In this study, the highest scoring item was “Follow your treatment regimen.” Of the participants, 445 (81.80%) chose “very confident” or “extremely confident,” and only 2 (0.37%) participants chose “not confident.” The lowest scoring item was “Evaluate changes in your blood pressure.” Of the patients, 41.91% had poor confidence in identifying changes in blood pressure. The results showed that older patients have higher self‐efficacy on treatment adherence, but they have difficulties when their blood pressure changes. However, compared with the results of Chen (2015) research, the self‐care confidence in this study was slightly lower. The possible reason is that elderly patients, whose body function, cognitive ability and state of mind were often worse than those of younger patients. Therefore, we should understand the physical and mental characteristics of elderly patients and promote their management of diseases proactively.

### 5.5 Analysis of predictors of self‐care and related interventions

#### 5.5.1 Predictors of self‐care and related interventions

In this study, we found that the predictors of self‐care among older adults with hypertension in China include age, family model, primary caregiver, maximum systolic blood pressure and receiving self‐care education. In this study, the older the participant was, the worse the self‐care was, as Haveman‐Nies et al. (2003) and Niriayo et al. (2019) reported. It indicates that medical staff should pay more attention to the high‐age elderly with hypertension. Family model refers to the members with whom the older adults live. The results showed that elderly hypertensive patients who lived alone had better self‐care, which may be related to solitude makes people more independent and self‐care aware, as Han et al. (2013) reported. On the contrary, the elderly who lived with spouse and children or with nanny had relatively poor self‐care, probably because they can get more care from others. The results showed that the elderly people who had people other than family members as the primary caregiver had better self‐care, probably because they become more independent, which is consistent with Han et al.’s (2013) research. Therefore, older adults should be encouraged to self‐care even if their primary caregivers are spouse or children. In this study, patients with higher maximum systolic blood pressure had better self‐care, which is consistent with Zhao et al.’s (2019) research. The possible reason is that these patients always have obvious symptoms and are more likely to notice the symptoms affecting their life quality and take self‐care measures. We found that elderly patients who often receive self‐care education had better self‐care, which is consistent with Ademe et al.’s (2019) research. It indicates that hypertension self‐care education should be carried out regularly.

Therefore, to enhance the self‐care of older adults with hypertension, medical staff should pay close attention to high‐age elderly, and give more considerate self‐care guidance. Self‐care should be encouraged for those living with family and have family members as primary caregivers.

#### 5.5.2 Predictors of subscale of self‐care and related interventions

In the subscale domain, we found that predictors of coverage of medical insurance, disease duration would influence self‐care maintenance, which is consistent with Niriayo et al.’s (2019) research. Predictors of age, maximum systolic blood pressure and receiving self‐care education would influence self‐care management, which is consistent with Ademe et al.’s (2019) research. Predictors of education level, economic burden, family model, disease duration, family history of hypertension and receiving self‐care education would influence self‐care confidence, which is consistent with Han et al. (2013), Ademe et al. (2019) and Lee and Park (2017) research.

Participants with higher education levels had better self‐care confidence, which is consistent with Feng et al. (2015), Koukouli et al. (2002) and Darrat et al. (2018) research. Highly educated patients often have better learning abilities, as Visanuyothin et al. (2018) reported that patients with good knowledge and literacy have a lower incidence of hypertension. Participants with family history of hypertension had better self‐care maintenance, which is consistent with Fan et al. (2010) research. The reason might be that these patients’ families have more awareness of disease‐related knowledge and give more care and supervision to patients.

The predictors mentioned earlier indicate that medical staff should pay more attention to the patients having full coverage of medical insurance and having long disease duration who have less self‐care maintenance. Moreover, patients with lower systolic blood pressure should strengthen blood pressure monitoring and timely detect blood pressure fluctuation. Health education should be provided through various forms to improve self‐care management. Patients having lower education level, heavy economic burden, family members as spouse and children, long disease duration and having no family history of hypertension often have lower level of self‐care confidence, who need to be paid special attention.

### 5.6 Limitations

In this study, 544 participants were all convenience sampled from one city in China due to the constraints of manpower, without fully considering the regional difference. We performed the preliminary analysis of the predictors of self‐care from the perspective of general information among older patients with hypertension, without full consideration of other factors. This study was a cross‐sectional study that we could not confirm the causal relationship between variables.

## 6 CONCLUSIONS

A cross‐sectional questionnaire survey was conducted in a convenience sampling of 544 older adults with hypertension using the Chinese version of SC‐HI. SPSS25.0 software was used for statistical analysis of the data. We found that the status of self‐care among older adults with hypertension in China is poor and needs more attention. The predictors from this study suggest which groups need special attention to improve self‐care and individualized intervention measures should be performed for older adults with hypertension.

## 7 RELEVANCE TO CLINICAL PRACTICE

In future study, we hope that multi‐regional and multi‐centre research can improve the representativeness of study results. It is necessary to expand the age stratification of the participants, considering factors including psychological, family and society, to explore more comprehensive predictors of self‐care. In addition, the action paths between variables should be explored on the basis of this study and experimental study should be carried out to identify the casual relationship between variables.

## CONFLICT OF INTEREST

The authors have no conflicts of interest to disclose.

## AUTHOR CONTRIBUTIONS

All authors have agreed on the final version and meet at least one of the following criteria [recommended by the ICMJE (http://www.icmje.org/recommendations/)]:

- substantial contributions to conception and design, acquisition of data or analysis and interpretation of data;
- drafting the article or revising it critically for important intellectual content.

## ETHICAL APPROVAL

This study was approved by the institutional review board of the Affiliated Hospital of Nantong University (Nantong University Ethical Review 2016‐K142).

## Supporting information

File S1

## Data Availability

The data that support the findings of this study are available from the corresponding author upon reasonable request.

## References

[nop21165-bib-0001] Ademe, S. , Aga, F. , & Gela, D. (2019). Hypertension self‐care practice and associated factors among patients in public health facilities of Dessie town, Ethiopia. BMC Health Services Research, 19(1), 51. 10.1186/s12913-019-3880-0 30665405 PMC6341627

[nop21165-bib-0002] Andreasen, N. C. , Arndt, S. , Cizadlo, T. , O'Leary, D. S. , Watkins, G. L. , Ponto, L. L. , & Hichwa, R. D. (1996). Sample size and statistical power in [15O]H_2_O studies of human cognition. Journal of Cerebral Blood Flow and Metabolism: Official Journal of the International Society of Cerebral Blood Flow and Metabolism, 16(5), 804–816. 10.1097/00004647-199609000-00005 8784225

[nop21165-bib-0003] Cao, Y. J. , Qi, S. F. , Yin, H. S. , Zhang, F. , Shi, W. W. , & Gao, J. C. (2019). Prevalence, awareness, treatment and control of hypertension in elderly residents in Hebei province. Chinese Journal of Epidemiology, 40(3), 296–300. 10.3760/cma.j.issn.0254-6450.2019.03.008 30884607

[nop21165-bib-0004] Chen, W. W. , Gao, R. L. , Liu, L. S. , Zhu, M. L. , Wang, W. , & Wang, Y. J. (2018). The report of cardiovascular disease in China 2018. Chinese Circulation Journal, 33(1), 1–8.

[nop21165-bib-0005] Chen, Y. E. (2015). Evaluation on reliability and validity of Chinese version of Hypertension Self‐care Profile (HBP SCP) and its preliminary application. Jiangsu University.

[nop21165-bib-0006] Chen, Y. , Cao, S. M. , & Yan, J. C. (2014). Reliability and validity of the Chinese‐version hypertension self‐care profile. Chinese Journal of Practical Nursing, 30(29), 12–16.

[nop21165-bib-0007] Chobanian, A. V. , Bakris, G. L. , Black, H. R. , Cushman, W. C. , Green, L. A. , Izzo, J. L. Jr , Jones, D. W. , Materson, B. J. , Oparil, S. , Wright, J. T. Jr , Roccella, E. J. & National High Blood Pressure Education Program Coordinating Committee . (2003). The seventh report of the joint national committee on prevention, detection, evaluation, and treatment of high blood pressure: The JNC 7 report. JAMA, 289(19), 2560–2572. 10.1001/jama.289.19.2560 12748199

[nop21165-bib-0008] Darrat, M. , Houlihan, A. , Gibson, I. , Rabbitt, M. , Flaherty, G. , & Sharif, F. (2018). Outcomes from a community‐based hypertension educational programme: The West of Ireland Hypertension study. Irish Journal of Medical Science, 187(3), 675–682. 10.1007/s11845-017-1706-9 29110187

[nop21165-bib-0009] Delavar, F. , Pashaeypoor, S. , & Negarandeh, R. (2019). The effects of self‐management education tailored to health literacy on medication adherence and blood pressure control among elderly people with primary hypertension: A randomized controlled trial. Patient Education and Counseling, 103(2), 336–342. 10.1016/j.pec.2019.08.028 31451361

[nop21165-bib-0010] Dickson, V. V. , Lee, C. , Yehle, K. S. , Abel, W. M. , & Riegel, B. (2017). Psychometric testing of the self‐care of hypertension inventory. The Journal of Cardiovascular Nursing, 32(5), 431–438. 10.1097/JCN.0000000000000364 27631117

[nop21165-bib-0011] Eckel, R. H. , Jakicic, J. M. , Ard, J. D. , de Jesus, J. M. , Houston Miller, N. , & Hubbard, V. S. , Lee, I.‐M. , Lichtenstein, A. H. , Loria, C. M. , Millen, B. E. , Nonas, C. A. , Sacks, F. M. , Smith, S. C. , Svetkey, L. P. , Wadden, T. A. , & Yanovski, S. Z. , & American College of Cardiology/American Heart Association Task Force on Practice Guidelines . (2014). 2013 AHA/ACC guideline on lifestyle management to reduce cardiovascular risk: a report of the American College of Cardiology/American Heart Association Task Force on Practice Guidelines. Journal of the American College of Cardiology, 63(25 Pt B), 2960–2984. 10.1016/j.jacc.2013.11.003 24239922

[nop21165-bib-0012] Fan, A. Z. , Mallawaarachchi, D. S. , Gilbertz, D. , Li, Y. , & Mokdad, A. H. (2010). Lifestyle behaviors and receipt of preventive health care services among hypertensive Americans aged 45 years or older in 2007. Preventive Medicine, 50(3), 138–142. 10.1016/j.ypmed.2009.12.007 20006640

[nop21165-bib-0013] Fan, W. G. , Xie, F. , Wan, Y. R. , Campbell, N. , & Su, H. (2020). The impact of changes in population blood pressure on hypertension prevalence and control in China. Journal of Clinical Hypertension, 22(2), 150–156. 10.1111/jch.13820 32003937 PMC8030006

[nop21165-bib-0014] Feng, Y. J. , Wang, H. C. , Li, Y. C. , & Zhao, W. H. (2015). Hypertension screening and follow‐up management by primary health care system among Chinese population aged 35 years and above. Biomedical and Environmental Sciences, 28(5), 330–340. 10.3967/bes2015.047 26055560

[nop21165-bib-0015] Han, H. R. , Lee, H. , Commodore‐Mensah, Y. , & Kim, M. (2014). Development and validation of the hypertension self‐care profile: A practical tool to measure hypertension self‐care. The Journal of Cardiovascular Nursing, 29(3), E11–E20. 10.1097/JCN.0b013e3182a3fd46 24088621 PMC3972381

[nop21165-bib-0016] Han, H. R. , Song, H. J. , Nguyen, T. , & Kim, M. T. (2014). Measuring self‐care in patients with hypertension: A systematic review of literature. The Journal of Cardiovascular Nursing, 29(1), 55–67. 10.1097/JCN.0b013e3182775fd1 23348221

[nop21165-bib-0017] Han, H. R. , Song, Y. , Song, H. J. , & Kim, M. T. (2013). Influence of living arrangements on the management and control of hypertension: A mixed‐methods study of Korean American elderly. Journal of Immigrant and Minority Health, 15(5), 944–952. 10.1007/s10903-012-9679-2 22790881 PMC3494797

[nop21165-bib-0018] Hansell, M. W. , Mann, E. M. , & Kirk, J. K. (2017). Hypertension treatment strategies for older adults. The Journal of Family Practice, 66(9), 546–554. 28863199

[nop21165-bib-0019] Haveman‐Nies, A. , De Groot, L. C. , Van Staveren, W. A. , & Survey in Europe on Nutrition and the Elderly: A Concerted Action Study . (2003). Relation of dietary quality, physical activity, and smoking habits to 10‐year changes in health status in older Europeans in the SENECA study. American Journal of Public Health, 93(2), 318–323. 10.2105/ajph.93.2.318 12554593 PMC1447737

[nop21165-bib-0020] Hypertension Branch of Chinese Association for Promotion of International Communication in Medical Care , Hypertension Professional Committee of Chinese Physicians Association , & Hypertension Branch of Chinese Gerontology Society . (2019). Chinese guidelines for prevention and treatment of hypertension (revised in 2018). Chinese Journal Cardiovascular Medicine, 24(1), 24.

[nop21165-bib-0021] Kim, M. T. , Hill, M. N. , Bone, L. R. , & Levine, D. M. (2000). Development and testing of the hill‐bone compliance to high blood pressure therapy scale. Progress in Cardiovascular Nursing, 15(3), 90–96. 10.1111/j.1751-7117.2000.tb00211.x 10951950

[nop21165-bib-0022] Koukouli, S. , Vlachonikolis, I. G. , & Philalithis, A. (2002). Socio‐demographic factors and self‐reported functional status: The significance of social support. BMC Health Services Research, 2(1), 20. 10.1186/1472-6963-2-20 12361478 PMC130039

[nop21165-bib-0023] Lee, E. , & Park, E. (2017). Self‐care behavior and related factors in older patients with uncontrolled hypertension. Contemporary Nurse, 53(6), 607–621. 10.1080/10376178.2017.1368401 28831843

[nop21165-bib-0024] Li, G. , Hu, H. , Dong, Z. , Xie, J. , & Zhou, Y. (2015). Urban and suburban differences in hypertension trends and self‐care: Three population‐based cross‐sectional studies from 2005–2011. PLoS One, 10(2), e0117999. 10.1371/journal.pone.0117999 25665069 PMC4322055

[nop21165-bib-0025] Li, Z. F. , Yang, H. , & Feng, X. X. (2014). A street survey about high‐salt diet‐related knowledge and its source. Journal of Changzhi Medical College, 28(2), 102–106.

[nop21165-bib-0026] Lu, J. , Lu, Y. , Wang, X. , Li, X. , Linderman, G. C. , Wu, C. , Cheng, X. , Mu, L. , Zhang, H. , Liu, J. , Su, M. , Zhao, H. , Spatz, E. S. , Spertus, J. A. , Masoudi, F. A. , Krumholz, H. M. , & Jiang, L. (2017). Prevalence, awareness, treatment, and control of hypertension in China: Data from 1·7 million adults in a population‐based screening study (China PEACE Million Persons Project). The Lancet, 390(10112), 2549–2558. 10.1016/S0140-6736(17)32478-9 29102084

[nop21165-bib-0027] Ma, X. , Zhang, Y. , Zhang, M. , Li, X. , Yin, H. , Li, K. , & Jing, M. (2019). Decomposing the effect of drug benefit program on antihypertensive medication adherence among the elderly in urban China. Patient Preference and Adherence, 13, 1111–1123. 10.2147/PPA.S201707 31371928 PMC6628968

[nop21165-bib-0028] Mahajan, S. , Zhang, D. , He, S. , Lu, Y. , Gupta, A. , Spatz, E. S. , Lu, J. , Huang, C. , Herrin, J. , Liu, S. , Yang, J. , Wu, C. , Cui, J. , Zhang, Q. , Li, X. , Nasir, K. , Zheng, X. , Krumholz, H. M. , Li, J. , … China PEACE Collaborative Group . (2019). Prevalence, awareness, and treatment of isolated diastolic hypertension: Insights from the China PEACE Million Persons Project. Journal of the American Heart Association, 8(19), e012954. 10.1161/JAHA.119.012954 31566101 PMC6806046

[nop21165-bib-0029] National Bureau of Statistics of China . (2020). Statistical Communique of The People’s Republic of China on the 2019 National Economic and Social Development. [EB/OL]. http://www.stats.gov.cn/tjsj/zxfb/202002/t202002281728913.html

[nop21165-bib-0030] Niriayo, Y. L. , Ibrahim, S. , Kassa, T. D. , Asgedom, S. W. , Atey, T. M. , Gidey, K. , Demoz, G. T. , & Kahsay, D. (2019). Practice and predictors of self‐care behaviors among ambulatory patients with hypertension in Ethiopia. PLoS One, 14(6), e0218947. 10.1371/journal.pone.0218947 31242265 PMC6594646

[nop21165-bib-0031] Or, C. , & Tao, D. (2016). A 3‐month randomized controlled pilot trial of a patient‐centered, computer‐based self‐monitoring system for the care of type 2 diabetes mellitus and hypertension. Journal of Medical Systems, 40, 81. 26802011 10.1007/s10916-016-0437-1

[nop21165-bib-0032] Orem, D. (2001). Nursing: Concepts and practice. Mosby.

[nop21165-bib-0033] Riegel, B. , & Dickson, V. V. (2008). A situation‐specific theory of heart failure self‐care. The Journal of Cardiovascular Nursing, 23(3), 190–196. 10.1097/01.JCN.0000305091.35259.85 18437059

[nop21165-bib-0034] Riegel, B. , Jaarsma, T. , & Strömberg, A. (2012). A middle‐range theory of self‐care of chronic illness. Advances in Nursing Science, 35(3), 194–204. 10.1097/ANS.0b013e318261b1ba 22739426

[nop21165-bib-0035] Silveira, L. , Rabelo‐Silva, E. R. , Ávila, C. W. , Beltrami Moreira, L. , Dickson, V. V. , & Riegel, B. (2018). Cross‐cultural adaptation of the self‐care of hypertension inventory into Brazilian Portuguese. The Journal of Cardiovascular Nursing, 33(3), 289–295. 10.1097/JCN.0000000000000442 28731915

[nop21165-bib-0036] Su, J. , Cui, L. , Du, W. C. , Miao, W. G. , Zhou, J. Y. , & Zhou, Y. L. (2019). Prevalence, awareness, treatment and control of hypertension in adult residents in Jiangsu province. Chinese Journal of Epidemiology, 40(9), 1139–1144. 10.3760/cma.j.issn.0254-6450.2019.09.023 31594161

[nop21165-bib-0037] Süderhamn, O. , Ek, A. C. , & Pürn, I. (1996). The self‐care ability scale for the elderly. Scandinavian Journal of Occupational Therapy, 3(2), 69–78. 10.3109/11038129609106686

[nop21165-bib-0038] Tabrizi, J. S. , Behghadami, M. A. , Saadati, M. , & Söderhamn, U. (2018). Self‐care ability of older people living in urban areas of Northwestern Iran. Iranian Journal of Public Health, 47(12), 1899–1905. 30788305 PMC6379604

[nop21165-bib-0039] Tang, H. Y. , Zhu, J. C. , He, H. Y. , Qian, C. R. , & Yang, Y. N. (2011). Behavioral characteristics of therapeutic adherence in patients with essential hypertension and factors influencing the adherence. Journal of Third Military Medical University, 33(16), 1747–1750.

[nop21165-bib-0040] Vaughan, D. V. , Lee, C. S. , Yehle, K. S. , Mola, A. , Faulkner, K. M. , & Riegel, B. (2017). Psychometric testing of the Self‐Care of Coronary Heart Disease Inventory (SC‐CHDI). Research in Nursing & Health, 40(1), 15–22. 10.1002/nur.21755 27686630

[nop21165-bib-0041] Villafuerte, F. U. , Cànaves, J. L. , Montalvo, P. L. , Sancho, M. L. M. , Oliver, B. O. , Flores, P. B. , Mantolan, A. E. , Bordoy, J. P. , Ruiz, T. R. , Hernández, A. R. , Leiva, A. , Quetglas, M. T. , Benejam, J. M. , Forteza, C. P. D. , Carratalà, F. R. , & The Medichy Group (2020). Effectiveness of a multifactorial intervention, consisting of self‐management of antihypertensive medication, self‐measurement of blood pressure, hypocaloric and low sodium diet, and physical exercise, in patients with uncontrolled hypertension taking 2 or more antihypertensive drugs. Medicine, 99(17), e19769. 10.1097/MD.0000000000019769 32332617 PMC7220514

[nop21165-bib-0042] Visanuyothin, S. , Plianbangchang, S. , & Somrongthong, R. (2018). Appearance and potential predictors of poorly controlled hypertension at the primary care level in an urban community. Journal of Multidisciplinary Healthcare, 11, 131–138. 10.2147/JMDH.S156518 29503561 PMC5826083

[nop21165-bib-0043] Wang, H. H. , & Laffrey, S. C. (2000). Preliminary development and testing of instruments to measure self‐care agency and social support of women in Taiwan. The Kaohsiung Journal of Medical Sciences, 16(9), 459–467. 11271731

[nop21165-bib-0044] Warren‐Findlow, J. , Basalik, D. W. , Dulin, M. , Tapp, H. , & Kuhn, L. (2013). Preliminary validation of the Hypertension Self‐Care Activity Level Effects (H‐SCALE) and clinical blood pressure among patients with hypertension. Journal of Clinical Hypertension, 15(9), 637–643. 10.1111/jch.12157 24034656 PMC8033917

[nop21165-bib-0045] Warren‐Findlow, J. , & Seymour, R. B. (2011). Prevalence rates of hypertension self‐care activities among African Americans. Journal of the National Medical Association, 103(6), 503–512. 10.1016/s0027-9684(15)30365-5 21830634 PMC3390197

[nop21165-bib-0046] WHO . (2002). Innovative care for chronic conditions. WHO.

[nop21165-bib-0047] Xin, C. , Zhang, B. , Fang, S. , & Zhou, J. (2020). Daytime napping and successful aging among older adults in China: A cross‐sectional study. BMC Geriatrics, 20(1), 2. 10.1186/s12877-019-1408-4 31898552 PMC6941277

[nop21165-bib-0048] Yan, X. F. , Yang, J. W. , Bai, X. K. , Wang, H. R. , Feng, F. , & Hou, L. L. (2020). Awareness, treatment and control of hypertension in 640 000 adults in eastern China. Chinese Journal of Epidemiology, 41(1), 68–73. 10.3760/cma.j.issn.0254-6450.2020.01.013 32062945

[nop21165-bib-0049] Zhao, Q. , Guo, Y. , Gu, Y. , & Yang, L. (2019). Translation and cross‐cultural adaptation of the Chinese version of the self‐care of hypertension inventory in older adults. The Journal of Cardiovascular Nursing, 34(2), 124–129. 10.1097/JCN.0000000000000522 30211817

